# Diltiazem improves contractile properties of skeletal muscle in dysferlin‐deficient BLAJ mice, but does not reduce contraction‐induced muscle damage

**DOI:** 10.14814/phy2.13727

**Published:** 2018-06-10

**Authors:** Morium Begam, Alyssa F. Collier, Amber L. Mueller, Renuka Roche, Sujay S. Galen, Joseph A. Roche

**Affiliations:** ^1^ Physical Therapy Program Department of Health Care Sciences Eugene Applebaum College of Pharmacy and Health Sciences Wayne State University Detroit Michigan; ^2^ Program in Physical Therapy Washington University in St. Louis School of Medicine St. Louis Missouri; ^3^ Program in Molecular Medicine University of Maryland School of Medicine Baltimore Maryland; ^4^ Eastern Michigan University School of Health Sciences Ypsilanti Michigan

**Keywords:** Diltiazem, dysferlin, eccentric contractions, LGMD2B, Miyoshi myopathy, muscle damage, skeletal muscle

## Abstract

B6.A‐*Dysf* ^*prmd*^/GeneJ (BLAJ) mice model human limb‐girdle muscular dystrophy 2B (LGMD2B), which is linked to mutations in the dysferlin (DYSF) gene. We tested the hypothesis that, the calcium ion (Ca^2+^) channel blocker diltiazem (DTZ), reduces contraction‐induced skeletal muscle damage, in BLAJ mice. We randomly assigned mice (*N* = 12; 3–4 month old males) to one of two groups – DTZ (*N* = 6) or vehicle (VEH, distilled water, *N* = 6). We conditioned mice with either DTZ or VEH for 1 week, after which, their tibialis anterior (TA) muscles were tested for contractile torque and susceptibility to injury from forced eccentric contractions. We continued dosing with DTZ or VEH for 3 days following eccentric contractions, and then studied torque recovery and muscle damage. We analyzed contractile torque before eccentric contractions, immediately after eccentric contractions, and at 3 days after eccentric contractions; and counted damaged fibers in the injured and uninjured TA muscles. We found that DTZ improved contractile torque before and immediately after forced eccentric contractions, but did not reduce delayed‐onset muscle damage that was observed at 3 days after eccentric contractions.

## Introduction

Dysferlin‐linked muscular dystrophies (a.k.a. dysferlinopathies) are a group of inherited diseases that cause progressive skeletal muscle weakness and wasting, with onset in the late‐teen years (Urtizberea et al. 2008; Harris et al. 2016). Dysferlinopathies are caused by mutations in the dysferlin (DYSF) gene, resulting in complete or partial absence of the protein dysferlin (Liu et al. 1998). Several theories have been proposed regarding the pathogenesis of dysferlinopathies, of which, one is, the presence of unstable transverse‐tubules (t‐tubules) and stress‐induced alterations in Ca^2+^ signaling in skeletal muscle (Bansal et al. 2003; Kesari et al. 2008; Nagaraju et al. 2008; Klinge et al. 2010; Kerr et al. 2013; Defour et al. 2014; Demonbreun et al. 2014; Hofhuis et al. 2017). The Ca^2+^ channel blocker diltiazem (DTZ), mitigates stress‐induced changes in Ca^2+^ signaling, and protects dysferlin‐deficient A/J mouse muscle fibers from stress‐induced cell damage, both in vitro and in vivo (Kerr et al. 2013). The in vivo data on the protective effect of DTZ against stress‐induced muscle fiber damage are of particular translational value, since dysferlin‐deficient A/J mice sustain severe delayed‐onset muscle damage, following injurious large‐strain eccentric contractions (large‐strain injury, LSI) to the tibialis anterior (TA) muscle (Roche et al. 2010, 2012, 2015). The data from A/J mice corroborate with the natural history of dysferlinopathies reported by patients, with regard to intense physical exercise and/or activity increasing muscle weakness (Angelini et al. 2011; Moore et al. 2018; Jain Foundation Inc., 2017). However, in addition to the dysferlin mutation, A/J mice carry several other mutations that might affect muscle physiology (Ho et al. 2004; The Jackson Laboratory, 2018). Furthermore, it has been reported that, dysferlin‐deficient B6.A‐*Dysf* ^*prmd*^/GeneJ (BLAJ) mice (developed by introgressing the A/J dysferlin mutation into the B6 mouse background) do not show as much delayed‐onset muscle damage as A/J mice, at 3 days after LSI (Lostal et al. 2010; Roche et al. 2012). Therefore, the LSI model is not ideal for preclinical screens of potential therapies for dysferlinopathy, in mouse strains other than A/J – such as the BLAJ strain (Roche et al. 2012). Furthermore, the LSI model involves rapidly moving the ankle joint of mice, from 90 to 180° of plantarflexion (1200°/sec), causing the foot to be almost in the same plane as the tibia at the end of the motion, raising concerns about the range of movement being nonphysiological and potentially injurious to the ankle‐foot complex. In order to address the issues described above, we developed a modified protocol of in vivo eccentric contractions, which involves plantarflexing the ankle from 90° to 160° (20° less plantarflexion than LSI), at an angular velocity of 300°/sec (25% the velocity of LSI). Also, instead of performing just one set of 20 repetitions as done in LSI, in our new eccentric contractions protocol, we perform 40 total repetitions, in four sets of 10 repetitions each, with 2 min rest between sets. Our new eccentric contractions protocol, referred to hereafter as Medium Strain Forced Eccentric Exercise (MSFEE, acronym pronounced “Ms. Fee”), is an eccentrically biased adaptation of a single bout in the MacQueen progressive resistance training regime (Macqueen 1954; Dutton 2011). The rationale for the increased number of reps in the MSFEE protocol compared to the LSI protocol, was based on pilot data, which suggested that, the 20 additional repetitions were required to consistently produce a similar loss of contractile torque as the LSI protocol (~ 50% of baseline torque), immediately after eccentric contractions in BLAJ mice (Roche et al. 2012). The studies described here, were designed to test the hypothesis that diltiazem protects dysferlin‐deficient BLAJ mouse muscle against contraction‐induced muscle damage.

## Methods

### Animal models

All experiments with live animals were performed at Wayne State University (Detroit, MI), according to protocols approved by the Institutional Animal Care and Use Committee. These protocols were in accordance with the Guide for the Care and Use of Laboratory Animals (1996, published by National Academy Press, 2101 Constitution Ave. NW, Washington, DC).

We studied dysferlin‐deficient, male, BLAJ mice (Stock No: 012767; The Jackson Laboratory, Bar Harbor, Maine, USA). The BLAJ strain of mice is a well‐characterized murine model of dysferlinopathy that resembles the human LGMD2B phenotype (Nagy et al. 2017). As the Jain Foundation Inc. (Bellevue, WA) maintains a colony of BLAJ mice at The Jackson Laboratory, the mice are provided as a gift from the Jain Foundation Inc. to partner laboratories.

We studied male mice, due to the availability of data from several published and pilot studies, on the response of control and dysferlin‐null male mice to injury by eccentric contractions (Roche et al. 2008, 2010, 2012, 2015; Millay et al. 2009b; Nagy et al. 2017). Mice were 3–4 months when we studied them, because at this age, BLAJ mice have a mature musculoskeletal system, but show very minimal spontaneous dystrophic changes in their skeletal muscles – this is an important consideration because spontaneous muscle damage might confound exercise‐induced muscle damage (Nagy et al. 2017).

We measured contractile torque, performed MSFEE, administered intraperitoneal injections of DTZ, and collected TA muscles, under inhaled isoflurane anesthesia (2–5% for induction; 1–4% for maintenance), in order to reduce handling stress and pain (Tabletop Anesthesia Machine, VetEquip, Livermore, CA). After collecting TA muscles, we euthanized mice by cervical dislocation (death ensured by bilateral pneumothorax) before proceeding to collect other tissues.

### Research design

We randomly assigned 12 mice to one of two study groups – namely, diltiazem‐treated group (DTZ, *N* = 6 mice) and a vehicle‐treated group (VEH, distilled water, *N* = 6 mice). We chose our sample sizes based on earlier studies, which suggested that, at Day 3 after eccentric contractions, in order for a 10‐percentage‐point reduction in the number of damaged fibers to be significant at an alpha level of 0.05 with a power of 0.80, we would need to study six animals per group.

We assigned a unique identification number for each mouse included in the study, and matched the number with an ear tagging and tail tattooing system. We conditioned mice with DTZ or VEH for 7 days before exposing them to MSFEE. On the day of MSFEE (Day 0), we assessed baseline contractile torque, exposed mice to the MSFEE protocol, studied immediate post‐MSFEE contractile torque, and returned animals to their original cages and then to the animal facility. We continued dosing with DTZ or VEH on the day of MSFEE and for 3 days, thereafter. On the third day post‐MSFEE (Day 3), we measured contractile torque to assess extent of recovery, and collected tissue per approved protocols (if MSFEE was performed on Monday, then Day 3 was Thursday). Details on how we dosed animals, studied contractile torque, performed MSFEE, processed tissue for histological studies, and quantified muscle damage, are described in specific subsections of the Methods.

### Contractile torque measurement and MSFEE

**Figure 1:**
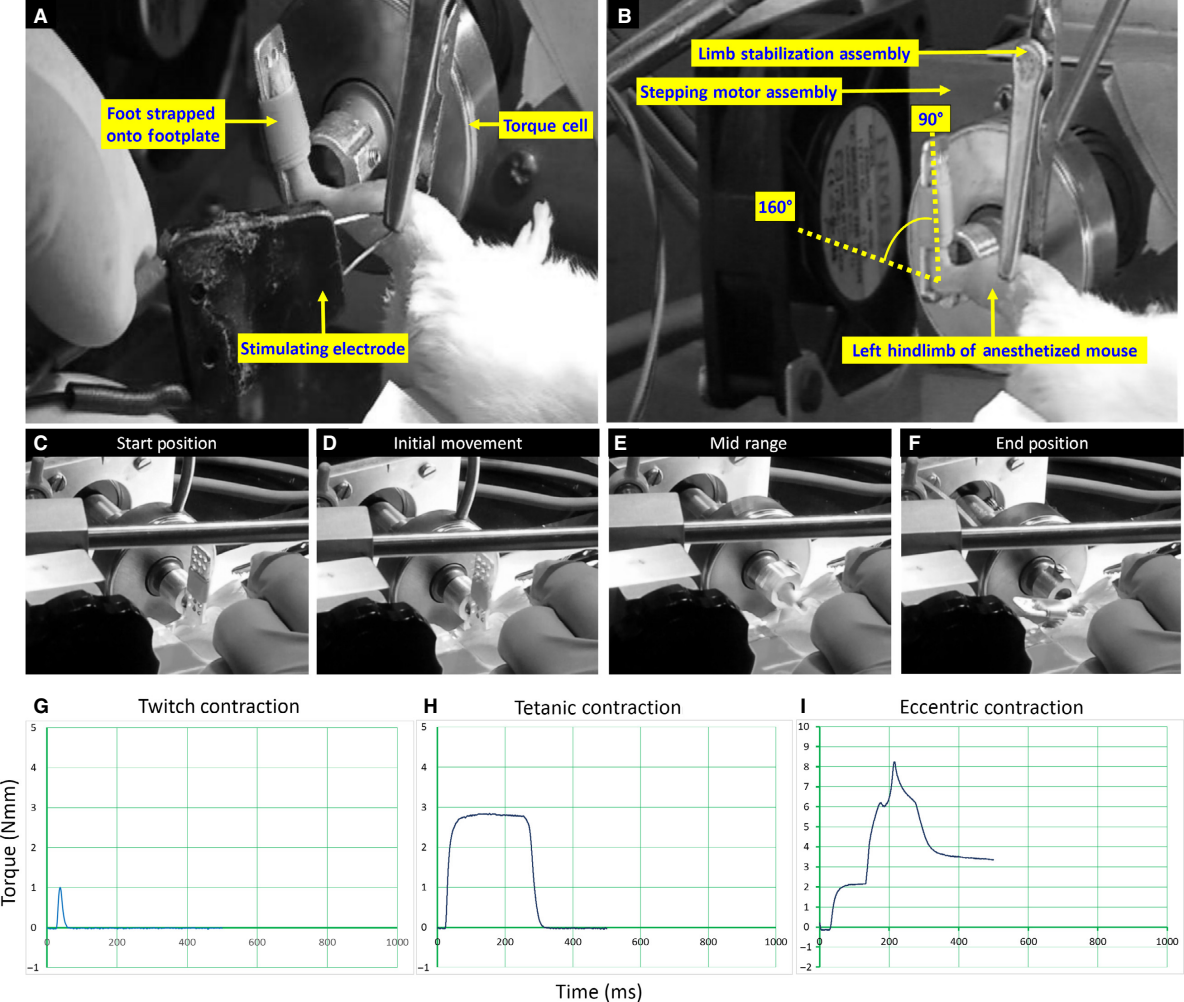
Setup for in vivo torque measurement and MSFEE. We measured contractile torque of the dorsiflexors of the left hindlimb and performed MSFEE on this muscle group with a custom‐built dynamometer (A and B). We elicited contractions of the dorsiflexors group of muscles, in which the prime mover is the TA muscle, by depolarizing the common peroneal nerve with a bipolar transcutaneous electrode (A). To perform forced eccentric contractions during MSFEE, we tetanically stimulated the dorsiflexors muscles, and forced the foot into plantarflexion (90–160°) with a stepper motor (B–F). We were able to trigger the electrical stimulator, control the motion of the stepper motor, and record torque data with custom‐written software. Representative torque traces for a single twitch, a maximal tetanic contraction, and a forced eccentric contraction, are shown in panels G, H, and I respectively. A single bout of MSFEE involved 40 eccentric contractions, performed in four sets of 10 repetitions, with 2 min rest between sets.

We measured contractile torque of the ankle dorsiflexors of the left hindlimb and exposed this muscle group to eccentrically biased forced exercise (MSFEE, 40 repetitions, 90–160° ankle plantarflexion during tetanic contraction of dorsiflexors). We measured contractile torque and performed MSFEE with a custom‐built rig, which is a scaled down version of an isokinetic dynamometer that is used for human testing and training (Bloch et al. 2012). Our rig is currently set up to study the ankle dorsiflexors of the left hindlimb of mice. As described in the Introduction, the MSFEE protocol is a refinement of the large‐strain injury (LSI) model that has been described and referenced in many publications (Roche et al. 2008, 2010, 2012, 2015; Millay et al. 2009b; Lovering et al. 2011; Lostal et al. 2012; Kerr et al. 2013). Images of our dynamometry rig are provided in Figure 1. We measured baseline contractile torque before MSFEE, the amount of torque lost immediately after MSFEE (Day 0, ~10 min post‐MSFEE), and the extent of torque recovery at 3 days post‐MSFEE (Day 3). We chose Day 3 for follow‐up studies, because delayed‐onset muscle damage from in vivo eccentric contractions peaks at this time point in dysferlin‐deficient A/J mouse muscle, and this phenotype provides a reliable set of outcome measures for preclinical testing of experimental therapies (Millay et al. 2009b; Roche et al. 2010, 2015; Lostal et al. 2012; Kerr et al. 2013).

Before measuring contractile torque or performing MSFEE, we removed fur from the animal's leg and lower thigh, and cleaned the area with three alternating scrubs of povidone iodine solution and 70% ethanol. We then passed a 26 G (1/2″) hypodermic needle (surgical steel) through the proximal tibial metaphysis (medial to lateral), removed the plastic hub of the needle, and held the ends of the needle in an alligator clamp, which was attached to a limb stabilization assembly on the dynamometry rig (Fig. 1A and B). We placed the animal in a supine position on the rig, and strapped its foot to a footplate that was linked in series to a torque transducer and a stepper motor (Fig. 1A–F). The movement of the stepper motor and acquisition of torque, were achieved through custom‐written routines in Labview 2014 (National Instruments, Austin, TX). We elicited contractions of the ankle dorsiflexors by electrically stimulating the common peroneal nerve with a transcutaneous bipolar electrode (BS4 50–6824, Harvard Apparatus, Holliston, MA) connected to an S48 square pulse stimulator (Grass Instruments, West Warwick, RI) through a PSIU6 stimulation isolation unit (Grass Instruments). We delivered pulses that were 0.1 msec in duration and adjusted the pulse amplitude in order to achieve maximal twitch torque in the ankle dorsiflexors (Fig. 1G). We used incremental pulse frequencies to generate a torque‐frequency curve. Maximal fused tetany occurred typically around 150 Hz, due to which, this stimulation frequency was used for eccentric contractions during MSFEE (Fig. 1H–I).

For each eccentric contraction during MSFEE, we induced a tetanic contraction in the ankle dorsiflexors, and superimposed a stretch on the dorsiflexors by passively plantarflexing the ankle with the stepper motor (90–160°) at an angular velocity of 300°/sec (Fig. 1C–F). For each set, 10 eccentric contractions were performed at ~1 contraction every 10 sec; and a total of four sets were performed with 2 min rest between sets.

The term “forced exercise” is applicable for exercise that is not performed voluntarily – such as, passive pedaling on a bicycle or similar experimental device (Houle et al. 1999; Ridgel et al. 2009; Beall et al. 2013). Therefore, the use of the term “Forced Eccentric Exercise” in MSFEE, is appropriate and accurate. We prefer the term “Forced Eccentric Exercise” to the term “Eccentric Injury”, because the MSFEE protocol follows a very specific set of parameters pertaining to the range of foot movement, the angular velocity of movement, the numbers of sets and repetitions of contractions, and the duration of rest between sets of contractions – all of which, are characteristic of an exercise protocol, and are in contrast to intentional injury models that involve eccentric contractions, mechanical impact, cryoinjury, or myotoxins (Lovering et al. 2005, 2007; Deane et al. 2014; Aguilar et al. 2015; Hardy et al. 2016). Furthermore, injury from MSFEE is a secondary consequence of forced exercise, and not the primary intent. It so happens that, several different murine models of muscular dystrophy sustain large amounts of acute or delayed‐onset muscle damage from forced eccentric contractions – examples: dystrophin‐deficient (mdx mouse model) muscle for acute muscle damage, and dysferlin‐deficient (A/J, BLAJ, SJL/J, B10.SJL mouse models) muscle for delayed‐onset muscle damage (Roche et al. 2012).

### Dosing with DTZ

We administered DTZ by intraperitoneal injection at a dosage of 72 mg/kg/day (Matsumura et al. 2009; Kerr et al. 2013). We dissolved 72 mg DTZ (Cat# 0685, Tocris, Minneapolis, MN) in 10 mL distilled water, vortex‐mixed the solution, and sterile‐filtered it. We aliquoted the sterile DTZ solution into 1.5 mL conical tubes and stored the tubes at −20°C until ready for use. Prior to injection, we allowed DTZ to thaw at 4°C, vortex‐mixed the solution, and injected 10 *μ*L/g (body weight) of each animal. We preconditioned animals for 7 days before MSFEE, because we have observed that, mice show increased sensitivity to inhaled isoflurane when given DTZ for the first time; incidentally, this also allows us to study the effects of short‐term dosing with DTZ on muscle physiology (Broadbent et al. 1985; Carceles et al. 1989; Drugs.com, 2018). On the day of MSFEE, we gave animals 60% of the daily dose ~15 min before MSFEE and 60% of the dose immediately after MSFEE. The split dose of DTZ, before and after MSFEE, was to reduce the risk of excessive cardiorespiratory depression during MSFEE under isoflurane anesthesia (Broadbent et al. 1985; Carceles et al. 1989; Drugs.com, 2018). Furthermore, since 86.4% mg/kg/day (120% of 72 mg/kg/day) is below the toxic dose of DTZ for mice, we administered 20% more DTZ than the daily dose, with the hope of mitigating acute muscle damage (Drugs.com, 2018). We administered sterile‐filtered distilled water alone to the VEH group, similar to how we administered DTZ.

### Histological studies

After collecting torque data at 3 days post‐MSFEE, we collected the exercised (left) and unexercised (right) tibialis anterior (TA) muscles, since the TA is the primary ankle dorsiflexor and the largest muscle in this group (Ingalls et al. 2004). We briefly dipped the muscles in mineral oil for cryoprotection, blotted off the excess oil with lab wipes, placed the muscles on pieces of aluminum foil, snap froze them by rapidly immersing them in liquid nitrogen, and stored them in cryogenic vials at −80°C.

To evaluate the general morphology of the TA and quantify myofiber damage, we performed hematoxylin and eosin (H&E) staining on 5 *μ*m cross sections as described (Roche et al. 2011, 2015; Begam et al. 2016). On additional TA muscle sections prepared as above, we assessed sarcolemmal and myofiber damage, respectively, based on the presence of mouse immunoglobulin G (IgG(+)) and loss of desmin labeling (desmin(−)) in the myoplasm of myofibers, as described (Begam et al. 2016).

We captured 12 digital images of each TA muscle cross‐section made from the mid‐belly of the muscle (20× objective). This method helped sample data across the cross‐section of an entire TA muscle by moving in a grid‐like fashion. We counted the total number of fibers (~2200 fibers per TA) and the number of damaged fibers for each muscle, and calculated the percentage of damaged fibers in each muscle (damaged fibers/total fibers*100).

### Statistical methods

We performed statistical analyses with IBM SPSS Statistics for Windows software (Version 25.0, IBM Corp. Armonk, NY). Since, our torque and histological data were not normally distributed, we performed nonparametric testing (Mann–Whitney *U* test) to ascertain if there were significant differences between the DTZ and VEH groups. For all statistical tests, the alpha level was set at 0.05, such that *P*‐values <0.05 were considered significant. All data are reported as mean ± standard deviation (SD). For raw pre‐MSFEE torque data and histological data, scatter‐plots of individual data points, are also shown. Group means and 95% confidence intervals (CIs) are presented as Supplementary Data.

## Results

### Contractile torque data

**Figure 2:**
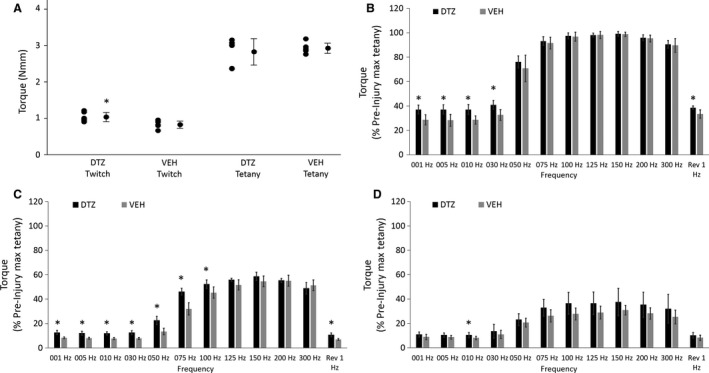
DTZ improves contractile torque before and immediately after MSFEE, but does not improve recovery at 3 days post‐MSFEE. DTZ increased baseline twitch torque (*), but did not increase baseline tetanic torque (A). Pre‐MSFEE torque‐frequency data suggest that, DTZ improves contractile torque at low frequencies (*, 1–30 Hz), but not at stimulation frequencies higher than 30 Hz (B). The frequency labeled reverse 1 Hz (Rev 1 Hz), is a twitch that is induced after the last tetanic contraction, in order to assess post‐tetanic changes in twitch torque (B–D). Torque data collected immediately after MSFEE suggest that, DTZ offers significant protection against acute torque loss from MSFEE (*, C). However, the protection against acute torque loss following MSFEE, is not seen at frequencies higher than 100 Hz (C). Torque data collected at 3 days post‐MSFEE suggest that, DTZ does not help improve torque recovery, except at the stimulation frequency of 10 Hz (D). Data are reported as mean ± SD. Asterisks denote a significant difference between DTZ and VEH.

Our baseline torque data (pre‐MSFEE torque data) indicate that, the DTZ group produced higher maximum twitch torque than the VEH group; however, there was no difference in maximum tetanic torque, between the DTZ and VEH groups (Fig. 2A). Our baseline torque‐frequency data indicate that, at low stimulation frequencies (1–30 Hz), there was a significant difference between the DTZ and VEH groups (Fig. 2B, Table S1).

The torque‐frequency data collected immediately after MSFEE indicate that, for stimulation frequencies up to 100 Hz, the DTZ group produced higher contractile torques (Fig. 2C, Table S2).

The torque‐frequency data collected at Day 3 post‐MSFEE indicate that, except for the 10 Hz stimulation frequency, there was no difference in contractile torque between the DTZ and VEH groups (Fig. 2D, Table S3).

### Histological data

**Figure 3:**
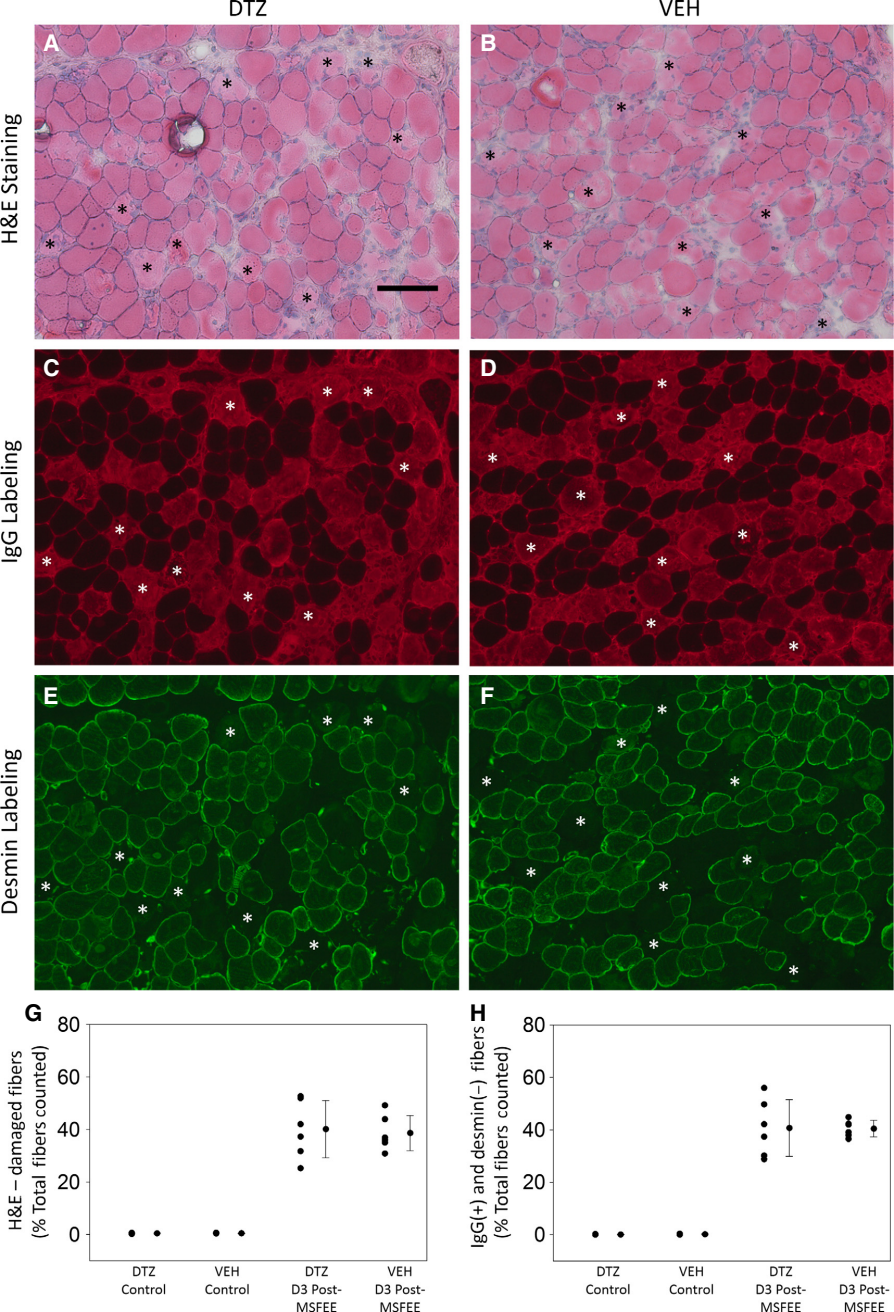
DTZ does not reduce delayed‐onset muscle damage at 3 days post‐MSFEE. Our data suggest that, extensive myofiber damage can be detected at 3 days post‐MSFEE in BLAJ mice, irrespective of whether the animals are given DTZ or VEH (A–F). This damage can be detected by the presence of myofibers with pale and/or disrupted cytoplasm in H&E stained sections (A and B), and by the inclusion of endogenous IgG (C and D) and loss of desmin (E and F) within myofibers. Since H&E staining, and IgG and desmin colabeling, were performed on serial cross‐sections of the same TA muscle; the superimposed asterisks (*) in the image panels, denote the same subset of damaged fibers in panels A, C, E (DTZ) and panels B, D, F (VEH). Scale bar = 100 *μ*m. Our quantitative analyses on images, such as, panels A–F, are shown in panels G (damaged fiber counts from H&E stained sections) and H (damaged fiber counts from IgG‐ and desmin‐labeled sections). Our data suggest that, at 3 days post‐MSFEE, DTZ does not reduce myofiber damage, as assessed by H&E staining (G) or IgG and desmin labeling (H). Data are reported as mean ± SD.

Representative images from the histological data that were studied, are presented in Fig. 3A–F. The data show extensive myofiber damage, which can be visualized through H&E staining (pale and disrupted cytoplasm of damaged myofibers, Fig. 3A and B), IgG labeling (IgG inclusion in damaged myofibers, Fig. 3C and D), and desmin labeling (loss of desmin in damaged myofibers, Fig. 3E and F). Since H&E staining, and IgG and desmin colabeling, were performed on serial cross‐sections of the same TA muscle, the same set of myofibers can be visualized in panels A, C, E (DTZ) and panels B, D, F (VEH) of Figure 3. The superimposed asterisks (*) in the image panels, denote the same subset of damaged fibers in serial sections (Fig. 3A, C, E, and B, D, F respectively).

Our quantitative analysis of images obtained from H&E staining, indicates that, there was no significant difference between DTZ and VEH groups, for muscle damage, at 3 days post‐MSFEE (Fig. 3G, Table S4). Likewise, our quantitative analysis of images obtained from and IgG and desmin colabeling, indicates that, there was no significant difference between DTZ and VEH groups, for muscle damage (IgG inclusion and desmin loss) at 3 days post‐MSFEE (Fig. 3H, Table S5).

## Discussion

We wanted to ascertain if the l‐type Ca^2+^ channel blocker DTZ, can reduce delayed‐onset muscle damage after eccentric contractions, in dysferlin‐deficient BLAJ mice. Our studies were a follow‐up to an earlier report, which suggested that, DTZ stabilized stress‐induced changes in t‐tubules and reduced delayed‐onset muscle damage in dysferlin‐deficient A/J mice (Kerr et al. 2013). In order to study BLAJ mice, we had to develop a new in vivo model of eccentric contractions, since the previously employed LSI model of eccentric contractions did not produce sufficient amounts of delayed‐onset muscle damage in dysferlinopathic mouse strains other than the A/J strain (Roche et al. 2012). We therefore developed the MSFEE protocol of eccentrically‐biased forced exercise, which uses parameters that are more functionally relevant than LSI (described in Introduction and Methods), and produces consistent delayed‐onset muscle damage of a magnitude that is a large enough to study the effects of DTZ in BLAJ mice.

Our torque data suggest that DTZ does improve baseline contractile function of BLAJ muscle. Specifically, torques at low stimulation frequencies (1–30 Hz), were higher in BLAJ mice treated with DTZ. However, DTZ did not improve the maximum tetanic torque (typically achieved at 125–150 Hz). These data suggest that DTZ is unlikely to improve maximum voluntary contraction (MVC) in patients with dysferlinopathies, and therefore not improve functional muscle strength (Horlings et al. 2008; Orr 2010; Shimada et al. 2010). We, however, did find that, DTZ offered significant protection against acute torque loss, soon after MSFEE (1–100 Hz), which might be of translational value; in relation to protection of dysferlin‐deficient from damage that might be induced by low‐intensity eccentric loading, during modified activities of daily living. At 3 days post‐MSFEE, where we hoped to see better torque production in the DTZ group than the VEH group, we did not see a significant difference in contractile torque for any of the stimulation frequencies except 10 Hz. These findings suggest that DTZ might be capable of producing some improvement in baseline contractile properties of dysferlinopathic muscle, and might even be able to protect muscle against acute torque loss from eccentric contractions, but that it does not promote faster recovery of contractile function at 3 days after injurious eccentric contractions.

Our histological data support the conclusions drawn from torque data collected at 3 days post‐MSFEE. We had predicted that DTZ would reduce the extent of delayed‐onset muscle damage observed at 3 days post‐MSFEE, similar to what we had seen after LSI in A/J mice. However, we observed that DTZ did not offer protection against muscle damage from MSFEE in BLAJ mice. Both DTZ and VEH groups had ~40% damaged myofibers in the exercised TA muscle, which validated the MSFEE protocol as suitable for preclinical studies of BLAJ mice, since it consistently produces extensive myofiber damage.

Our current studies serve to temper earlier conclusions, drawn from studies on dysferlin‐deficient A/J mice, for the protective effects of DTZ on dysferlinopathic muscle (Kerr et al. 2013). As concluded in an earlier study, our current studies support the notion that, the pathology dysferlin‐deficiency might be much more complex than a simple failure of sarcolemmal resealing or t‐tubule instability, even though these are salient features of dysferlin‐deficient muscle cells (Lostal et al. 2012). Furthermore, even though DTZ might confer some benefit on dysferlinopathic muscle; the recent concerns about calcium channel blockers being associated with an increased incidence of mood disorders, must be taken into consideration, if and when calcium channel blockers are prescribed as a pharmacological intervention for dysferlinopathies (Boal et al. 2016).

Our studies also serve to emphasize the value of muscle‐injury‐prevention in patients with dysferlinopathies. Since a large percentage of patients with dysferlinopathies are known to have athletic prowess during the years prior to onset of muscle weakness, it is not uncommon for patients to exercise more, when they start developing symptoms (Harris et al. 2016; Moore et al. 2018; Jain Foundation Inc., 2017). The evidence so far suggests that, like most other muscular dystrophies (other than some congenital muscular dystrophies), dysferlinopathies are progressive conditions, and therefore, cannot be reversed by exercise. The fact that dysferlinopathies are progressive conditions, should encourage the focus of rehabilitative interventions, to be more on slowing down the natural progression of muscle weakness and wasting, rather than increasing muscle strength and walking capacity at the risk of causing irreversible muscle damage. Preserving functional muscle mass for as long as possible, preventing joint contractures, and maintaining the central nervous system's synaptic connections for functional movement, might help optimize patient‐readiness for promising gene therapies that are under intense investigation (Lostal et al. 2010; Long et al. 2014; Sondergaard et al. 2015; Nelson et al. 2016; Robinson‐Hamm and Gersbach 2016; Tabebordbar et al. 2016; Benedetti et al. 2017). In the context of our current set of studies, it appears that, DTZ is not likely to reduce the damage that is induced by a moderately intense bout of repetitive eccentric loading, in dysferlin‐deficient mammalian skeletal muscle. Therefore, even if a patient with dysferlinopathy takes DTZ, it would still be important to choose muscle contractions (example: concentric vs. eccentric) and exercise loads (example: low repetition vs. high repetition) to obtain the benefits of exercise without causing extensive muscle damage (Roche et al. 2010; Biondi et al. 2013).

The translational significance of our findings is that, DTZ might be able to improve baseline contractile properties of skeletal muscle in patients with dysferlinopathies, but might not be able to mitigate contraction‐induced muscle damage from a bout of moderately intense eccentric contractions. Therefore, even if patients with dysferlinopathies take a calcium channel blocker like DTZ for cardiovascular comorbidities and/or for improved muscle function; they are likely to continue to be at risk for developing contraction‐induced muscle damage, if they perform activities that involve intense and repetitive eccentric muscle loading (Biondi et al. 2013).

Even though our current study focused on the potential beneficial effects of DTZ on dysferlin‐linked muscular dystrophy, our findings might also be of relevance to other muscular dystrophies (Phillips and Quinlivan 2008; Matsumura et al. 2009). Although the primary pathological bases for progressive muscle weakness and wasting differ between different types of muscular dystrophy; there is consensus that, irrespective of the type of muscular dystrophy, myofiber death is ultimately linked to elevated Ca^2+^ levels in the cytoplasm of muscle fibers (Millay et al. 2009a; Burr and Molkentin 2015). Therefore, the prediction would be that, blocking the entry of Ca^2+^ from the extracellular environment into the myoplasm with DTZ, should reduce the dystrophic phenotype in other muscular dystrophies (example: Duchenne muscular dystrophy). However, despite promising preclinical results, clinical data suggest that DTZ does not significantly alter functional outcomes in patients with Duchenne muscular dystrophy (Phillips and Quinlivan 2008; Matsumura et al. 2009). Similar to our discussion, above, on the role of exercise for dysferlinopathies; it is likely that, the pathological processes underlying muscular dystrophies are too severe, for drugs like DTZ, to significantly alter functional outcomes in clinical trials. However, pharmacological agents that effectively inhibit dystrophic muscle loss in preclinical models, might still have a role in preserving muscle mass, and optimizing patient‐readiness for promising gene therapies, even if they don't significantly improve standard functional outcomes (example: 6‐min walk test, timed up and go test, etc.). Single case studies on patients with dysferlinopathies who have been prescribed DTZ, might provide insights into whether large clinical trials are warranted.

## Conclusion

Diltiazem improves contractile properties of dysferlin‐deficient BLAJ mouse muscle, after 7 days of dosing. Diltiazem also reduces the acute loss of contractile torque immediately after eccentric contractions. However, diltiazem does not reduce delayed‐onset muscle damage observed at 3 days after eccentric contractions.

## Conflict of Interest

The authors have no conflicts to declare.

## Data Accessibility

## Supporting information




**Table S1.** DTZ versus VEH preinjury torque.
**Table S2.** DTZ versus VEH immediate postinjury torque.
**Table S3.** DTZ versus VEH day 3 postinjury torque.
**Table S4.** H&E ‐ damaged fibers (% total).
**Table S5.** IgG (+) and desmin (−) fibers (% total).
