# Supplementary material for: Diltiazem improves contractile properties of skeletal muscle in dysferlin‐deficient BLAJ mice, but does not reduce contraction‐induced muscle damage
Source: Physiol Rep. 2018 Jun 10;6(11):e13727. doi: 10.14814/phy2.13727 (PMC5995314; doi:10.14814/phy2.13727)
Supplement: Supplementary file 1 — Table S1. DTZ versus VEH preinjury torque. Table S2. DTZ versus VEH immediate postinjury torque. Table S3. DTZ versus VEH day 3 postinjury torque. Table S4. H&E ‐ damaged fibers (% total). Table S5. IgG (+) and desmin (−) fibers (% total). [file PHY2-6-e13727-s001.docx]

| **SUPPLEMENTARY DATA. TABLE S1. DTZ Vs VEH Pre-Injury Torque** | | | | |
| --- | --- | --- | --- | --- |
|  | **DTZ Mean**  **(95% CI)** | **VEH Mean**  **(95% CI)** | **P value** | **Significant** |
| **1 Hz** | 37  (33.13, 40.87) | 28.5  (24.11, 32.89) | 0.004 | YES |
| **5 Hz** | 37  (32.80, 41.20) | 28.17  (23.05, 33.28) | 0.009 | YES |
| **10 Hz** | 37.17  (32.79, 41.54) | 28.5  (24.94, 32.06) | 0.004 | YES |
| **30 Hz** | 41  (37.13, 44.87) | 32.67  (28.04, 37.3) | 0.004 | YES |
| **50 Hz** | 70.83  (70.51, 81.16) | 70.83  (59.37, 82.28) | 0.589 | NO |
| **75 Hz** | 93.17  (89.45, 96.87) | 91.5  (86.41, 96.59) | 0.589 | NO |
| **100 Hz** | 97.5  (95.13, 99.87) | 96.83  (93.05, 100.61) | 0.937 | NO |
| **125 Hz** | 98.17  (96.62, 99.71) | 98.17  (95.24, 101.09) | 0.589 | NO |
| **150 Hz** | 99.17  (97.49, 100.85) | 98.83  (97.15, 100.51) | 0.699 | NO |
| **200 Hz** | 95.83  (93.4, 98.26) | 95.33  (92.54, 98.12) | 0.589 | NO |
| **300 Hz** | 90.5  (87.07, 93.93) | 89.83  (83.7, 95.97) | 0.589 | NO |
| **Rev 1Hz** | 38.67  (37.4, 39.94) | 33.17  (29.39, 36.95) | 0.009 | YES |

| **SUPPLEMENTARY DATA. TABLE S2. DTZ Vs VEH Immediate Post-Injury Torque** | | | | |
| --- | --- | --- | --- | --- |
|  | **DTZ Mean**  **(95% CI)** | **VEH Mean**  **(95% CI)** | **P value** | **Significant** |
| **1 Hz** | 12.67  (11.08, 14.25) | 8.17  (7.38, 8.96) | 0.002 | YES |
| **5 Hz** | 12.17  (10.36, 13.97) | 7.83  (6.8, 8.87) | 0.004 | YES |
| **10 Hz** | 11.83  (10.29, 13.38) | 7.5  (6.62, 8.38) | 0.002 | YES |
| **30 Hz** | 12.5  (10.77, 14.22) | 7.67  (6.81, 8.52) | 0.002 | YES |
| **50 Hz** | 22.33  (18.78, 25.89) | 13.67  (10.96, 16.38) | 0.002 | YES |
| **75 Hz** | 46.17  (43.10, 49.24) | 31.67  (26.5, 36.84) | 0.002 | YES |
| **100 Hz** | 52.33  (48.84, 55.82) | 45.17  (40.14, 50.2) | 0.015 | YES |
| **125 Hz** | 56  (54.51, 57.48) | 51.83  (47.26, 56.40) | 0.132 | NO |
| **150 Hz** | 58.67  (55.11, 62.22) | 54.5  (49.91, 59.09) | 0.132 | NO |
| **200 Hz** | 55.33  (53.62, 57.05) | 55.17  (50.5, 59.83) | 0.937 | NO |
| **300 Hz** | 48.83  (43.80, 53.86) | 51.33  (46.47, 56.2) | 0.818 | NO |
| **Rev 1Hz** | 10.67  (9.23, 12.1) | 7  (6.34, 7.66) | 0.002 | YES |

| **SUPPLEMENTARY DATA. TABLE S3. DTZ Vs VEH Day 3 Post-Injury Torque** | | | | |
| --- | --- | --- | --- | --- |
|  | **DTZ Mean**  **(95% CI)** | **VEH Mean**  **(95% CI)** | **P value** | **Significant** |
| **1 Hz** | 11  (8.80, 13.20) | 8.67  (6.4, 10.93) | 0.180 | NO |
| **5 Hz** | 10.33  (8.75, 11.91) | 8.67  (7.09, 10.25) | 0.180 | NO |
| **10 Hz** | 10.33  (8.27, 12.40) | 8  (6.67, 9.33) | 0.026 | YES |
| **30 Hz** | 13.33  (7.19, 19.48) | 11  (7.75, 14.25) | 0.818 | NO |
| **50 Hz** | 23.17  (18.18, 28.15) | 20.5  (16.70, 24.3) | 0.394 | NO |
| **75 Hz** | 32.67  (25.36, 39.35) | 26.17  (21, 31.33) | 0.132 | NO |
| **100 Hz** | 36.5  (27.10, 45.90) | 27.83  (22.89, 32.77) | 0.065 | NO |
| **125 Hz** | 36.5  (26.96, 46.04) | 29  (23.65, 34.35) | 0.093 | NO |
| **150 Hz** | 37.5  (25.95, 49.05) | 31  (26.96, 35.04) | 0.180 | NO |
| **200 Hz** | 35.17  (24.33, 46) | 28.17  (23.41, 32.93) | 0.240 | NO |
| **300 Hz** | 31.83  (19.5, 44.17) | 25.33  (19.48, 31.18) | 0.310 | NO |
| **Rev 1Hz** | 10.17  (7.83, 12.51) | 8.33  (5.97, 10.7) | 0.310 | NO |

| **SUPPLEMENTARY DATA. TABLE S4. H&E - Damaged Fibers (% Total)** | | | | |
| --- | --- | --- | --- | --- |
|  | **DTZ Mean**  **(95% CI)** | **VEH Mean**  **(95% CI)** | **P value** | **Significant** |
| **Uninjured** | 0.37  (0.18, 0.55) | 0.41  (0.29, 0.52) | 0.75 | NO |
| **Injured** | 40.13  (28.62, 51.63) | 38.64  (31.61, 45.66) | 0.63 | NO |

| **SUPPLEMENTARY DATA. TABLE S5. IgG (+) and Desmin (-) Fibers (% Total)** | | | | |
| --- | --- | --- | --- | --- |
|  | **DTZ Mean**  **(95% CI)** | **VEH Mean**  **(95% CI)** | **P value** | **Significant** |
| **Uninjured** | 0.10  (0, 0.21) | 0.20  (0.2, 0.38) | 0.52 | NO |
| **Injured** | 40.75  (29.5, 52.04) | 40.5  (37.23, 43.76) | 0.87 | NO |
